# Construction of PCR-SERS Method for Detection of *Vibrio parahaemolyticus*

**DOI:** 10.3390/foods13111743

**Published:** 2024-06-01

**Authors:** Antuo Hu, Xiaoting Song, Xiaojie Sun, Zhaoxin Lu, Xinmei Liu, Xiaomei Bie, Jun Yang

**Affiliations:** 1 College of Food Science and Technology, Nanjing Agricultural University, Nanjing 210095, China; 2019208013@stu.njau.edu.cn (A.H.); 2022108046@stu.njau.edu.cn (X.S.); fmb@njau.edu.cn (Z.L.); 2 Key Laboratory of Detection and Traceability Technology of Foodborne Pathogenic Bacteria for Jiangsu Province Market Regulation, Nanjing Institute for Food and Drug Control, Nanjing 211198, China; sxj6447813@163.com (X.S.); 18951782919@163.com (X.L.)

**Keywords:** *Vibrio parahaemolyticus*, surface-enhanced Raman scattering (SERS), polymerase chain reaction (PCR), Eva Green

## Abstract

A paper-based surface enhancement of a Raman scattering substrate consisting of silver-nanowires stacked on glass-fiber filter paper was prepared. At the same time, the DNA-embedding molecule Eva Green was introduced as a signaling molecule for surface-enhanced Raman scattering (SERS) detection. Polymerase chain reaction (PCR) was used to amplify target genes and the method was developed into a rapid molecular diagnostic system. The total detection time of the developed detection method was 40 min, including 30 min of PCR amplification and 10 min of SERS measurement. After 30 PCR cycles, bacterial DNA with an initial concentration of 20 fg/μL and a bacterial suspension with an initial concentration of 7.2 × 10^1^ CFUs/mL could be detected. When the enrichment culture time was 4 h, target bacteria with an initial contamination inoculation volume of 1.5 CFUs/mL could be detected in artificially contaminated samples. The method is fast and highly sensitive, and has not been applied to the detection of *V. parahaemolyticus.*

## 1. Introduction

Raman spectroscopy is a scattering spectrum based on the Raman scattering effect. A light beam is irradiated onto the object to be detected, and the photons interact with the object to be detected to generate scattered light [1]. The structure and composition of a substance are reflected by measuring the characteristic frequency shift of molecular vibration or rotational energy levels. Therefore, Raman spectroscopy is also called the molecular “fingerprint” spectrum [2]. Raman scattering is an inelastic scattering phenomenon caused by light striking the surface of a material. When the incident light photons of the monochromatic beam interact with the molecules of the object to be detected, elastic collisions and inelastic collisions can occur at the same time. During the elastic collision process, the direction of movement of the photons changes but the frequency remains unchanged, and this scattering is called Rayleigh scattering [3]. During the inelastic collision process, the photon exchanges energy with the detected molecule, thereby changing the frequency of the photon, and this scattering is called Raman scattering [4]. In 1974, Fleischmann et al. discovered that pyridine molecules adsorbed on a rough Ag electrode surface had obvious Raman scattering effects, and the surface-enhanced Raman spectroscopy (SERS) technology was born [5,6].

SERS substrates are generally divided into metal substrates, semiconductor material substrates, and nanocomposite substrates according to the complexity of their material composition [7]. Among them, the detection sensitivity of nanocomposite substrates is relatively high, but the preparation steps are cumbersome. The preparation of single-metal substrates is simple, but the sensitivity is slightly insufficient. One-dimensional nanostructures such as nanowires are important members of the nanomaterial family and have been widely used in research fields such as nanooptics, molecular sensing, and biology [8]. Silver nanowires (AgNWs) are some of the nanomaterials and have become one of the most studied nanowires due to their promising mechanical, optical and electronic properties [9]. So far, AgNWs have been used as plasmonic waveguides, SERS platforms, for single-cell endoscopy, as cutting-edge enhanced spectroscopy probes, etc. [10,11,12,13]. Silver nanowires combined with SERS have been successfully used in the detection of microorganisms. For example, Ankudze et al. proposed a simple method based on hydraulic pressure to obtain AgNW–cotton fiber materials, using 3-aminopropyltrimethoxysilane as a connecting agent to fix AgNWs on cotton fibers, and compressed them by a hydraulic press to reduce the free space between individual fibers; the prepared substrate could be used to effectively filter *E. coli* in phosphate buffer and urine, and was successfully applied for SERS detection. Therefore, silver nanowire–cotton fibers can be used as a low-cost SERS platform for the effective detection of bacteria in liquids [14].

SERS has great potential in DNA detection. Macdonald et al. used Raman tag-labeled silver nanoparticles to directionally identify target DNA molecules and provide enhanced Raman signals [15]. In order to reduce the thermal cycling steps and shorten the detection time, Wu et al. developed a SERS-PCR detection method using AuNPs, which can detect DNA with an initial concentration of 1 × 10^5^ copies/μL in eight PCR cycles [16]. Similarly, Lee et al. combined a paper-based SERS substrate composed of silver nanowires with PCR to quickly and sensitively detect respiratory bacterial DNA. After 10 cycles, the PCR-SERS method showed enhanced detection capabilities, with a DNA detection limit of 3.12 pg/μL [17].

*Vibrio parahaemolyticus* (*V. parahaemolyticus*) is a human pathogen that is widely distributed in the marine environment and is often isolated from various raw seafood. Consumption of raw or undercooked seafood products contaminated with *V. parahaemolyticus* may cause food poisoning and lead to the development of acute gastroenteritis. Although gastroenteritis caused by *V. parahaemolyticus* is usually self-limiting, it can be life-threatening in people with underlying medical conditions such as liver disease or immune disorders. Currently, the most common and easy detection methods for *V. parahaemolyticus* are molecular biology methods based on nucleic acid amplification. The accuracy and specificity of such methods mainly depend on the selection of detection targets. This team used bioinformatics methods to discover new detection targets for *V. parahaemolyticus*. Based on previous work, we selected five groups of target genes and constructed a PCR-SERS method [18].

This study first prepared a simple and efficient detection substrate, and combined SERS technology with PCR. The primer dependency of PCR technology is used to solve the need for the preparation of SERS-specific probes. On the one hand, it saves costs, and on the other hand, it ensures the specificity of the detection method. SERS probes have the problem of a low capture rate, and the problem can be avoided by using the specificity of the detection target and the specificity of PCR primers. At the same time, the signal amplification function of SERS allows signal changes caused by trace amounts of DNA to be detected. Therefore, SERS technology can perform ultra-sensitive molecular detection, reducing the detection time and improving detection sensitivity. Combining SERS with PCR technology is expected to create a highly specific, rapid, and sensitive detection method.

## 2. Materials and Methods

### 2.1. Materials

#### 2.1.1. Bacterial Strains and Culture Conditions

The *Vibrio parahaemolyticus* isolates used in this experiment are strains stored in our laboratory. Among the standard strains, the ATCC strain was purchased from the American Type Culture Collection (ATCC), the CICC strain was purchased from China Center of Industrial Culture Collection (CICC), the CGMCC strain was purchased from China General Microbiological Culture Collection Center (CGMCC), the CMCC strain was purchased from China Medical Bacteria National Center for Medical Culture Collections (CMCC), and MCCC strains were purchased from Marine Culture Collection of China (MCCC).

#### 2.1.2. Main Reagents

The paper-based substrate is composed of glass-fiber filter paper (GFP) and AgNWs. The glass-fiber filter paper and filter membrane holder were purchased from Whatman Company (Maidstone, UK). The silver nanowires were purchased from Jiangsu Xianfeng Nano Materials Technology Co., Ltd. (Nanjing, China).

### 2.2. Methods

#### 2.2.1. PCR Method

Strain ATCC 17802 was cultured and activated using 3% NaCl TSA solid medium overnight at 37 °C, and then secondarily activated using 3% NaCl TSB liquid medium overnight at 37 °C. The genomic DNA of the strain was extracted by a thermal lysis method and diluted 10 times, 100 times and 1000 times, respectively, to be used as a template for subsequent primer screening. The 20 μL PCR system included 2 μL of 10× Ex Taq PCR buffer, 0.8 μL of dNTP mixture (2.5 mM), 0.1 μL of Ex Taq HS enzyme, 1 μL each of upstream and downstream primers (F/R), and 2 μL of DNA template, and was made up to 20 μL with ddH_2_O. The PCR procedure adopted a two-step method: pre-denaturation at 95 °C for 30 s, and the cycle program was denaturation at 95 °C for 10 s and annealing and extension at 60 °C for 30 s, for a total of 30 cycles. PCR products were electrophoresed using a 1.5% agarose gel at 140 V for 30 min. In the primer determination stage, 5 targets were selected: *VP0091*, *VP0289*, *VP0249*, *VP0488* and *VP0811*. The primer information is shown in Table 1.

#### 2.2.2. Construction and Characterization of SERS Substrates

During the detection substrate construction phase, correctly assemble the GFP and filter membrane holder, add 1.5 mL of the AgNW solution with a concentration of 10 mg/mL into the filter membrane holder through the sample port, and use a vacuum filtration device for suction filtration. After the suction filtration is complete, the filter paper is completely removed and placed in an oven to dry at 70 °C for 20 min to obtain a SERS paper-based substrate. The constructed paper-based substrates were subjected to microscopic observation and appearance characterization using scanning electron microscopy (Phenom Pure G6 Desktop SEM, Thermo Fisher Scientific Co., Ltd., Waltham, MA, USA).

#### 2.2.3. Establishment of Detection Methods

When performing the test, cut the prepared detection substrate into small pieces of 5 mm × 5 mm, add 10 μL of the sample to be tested to each small piece, and dry it in an oven at 60 °C for 5 min. SERS detection was performed using a Thermo Fisher DXR 2xi microscope Raman imaging spectrometer (Thermo Fisher Scientific Co., Ltd., Waltham, MA, USA).

In order to verify the feasibility of the method, the traditional Raman spectrum signaling molecule rhodamine B (Rhod B) was first used in the experiment, and a total of 8 concentration gradients were set: 1 μM, 500 nM, 100 nM, 50 nM, 25 nM, 10 nM, and 1 nM. The Raman signal response values were observed for different signaling molecule concentrations. The exposure time, laser power, and laser wavelength in the detection conditions used were 0.02 s, 3 mW, and 633 nm, respectively.

Since there is a negative correlation between the template concentration and Raman signal, in order to facilitate the determination of detection results, the ΔRaman intensity value (ΔRI value) was calculated according to the following method: ΔRI = RI_substrate_ − RI_sample_, in which RI_substrate_ is the average signal value of the detection substrate, and RI_sample_ is the Raman value reported at the Raman shift of 1374 cm^−1^ for the samples to be tested.

#### 2.2.4. Screening of Signaling Molecules

First, Eva Green was selected as a signaling molecule, and its effective range of use and ability as a signaling molecule were evaluated. According to preliminary experiments, similar DNA chimeric dyes include Syto Green 9, Cybr Green, LY Green, and DNA Green. In subsequent experiments, the types and concentrations of signaling molecules were screened.

#### 2.2.5. Optimization of the PCR Amplification Time

In this part of the experiment, the detection limit of the method was measured at 10, 20, and 30 PCR cycles. The final amplification time was determined based on the detection capabilities of the method at different cycle numbers.

#### 2.2.6. Method Sensitivity Assessment

The sensitivity of the method was tested using the *V. parahaemolyticus* standard strain ATCC 17802. When performing DNA template sensitivity testing, DNA templates with a concentration range of 20 ng/μL–2 fg/μL were amplified by PCR and then subjected to SERS detection to evaluate the DNA template sensitivity of the method. The plate count of the cultured bacterial suspension was 7.2 × 10^8^ CFUs/mL. After gradient dilution, a bacterial liquid template of 7.2–7.2 × 10^8^ CFUs/mL was obtained, and the DNA was extracted by the thermal lysis method to evaluate the bacterial suspension sensitivity of this method.

#### 2.2.7. Method Specificity Assessment

The method specificity was evaluated using 9 standard strains of *V. parahaemolyticus*, 9 strains of *V. parahaemolyticus* isolates, and 20 negative strains. Negative strains mainly included common foodborne pathogens and non-*V. parahaemolyticus* strains, which are also members of the genus *Vibrio*.

#### 2.2.8. Artificially Contaminated Sample Detection

Artificially contaminated samples were prepared using sea shrimp. First, the shrimp samples were washed with sterile saline three times and then cut into small pieces on a clean workbench. A total of 5 g of sample was ground and mixed with 20 mL of sterile PBS buffer to form a shrimp homogenate. The mixture was filtered through a 0.22 μm filter membrane and inoculated with serially diluted *V. parahaemolyticus*. A total of 1 mL of the homogenate sample was taken every two hours for DNA extraction, and templates were collected from 0 to 8 h for detection using the established method.

#### 2.2.9. Determination of the Actual Sample Recovery Rate

Samples of oysters, clams, cod, and river shrimp were purchased from the Nanjing Seafood Market. After the same treatment as in Section 2.2.8, each sample was inoculated with *V. parahaemolyticus* (the concentration measured by the plate counting method was 500 CFUs/mL), and the recovery rate was calculated to verify the practicability and quantitative detection capability of the method.

#### 2.2.10. Data Processing

All measurement data in this article were repeated three times, and the processed data were used to generate line charts or column charts using GraphPad Prism 9.

## 3. Results

### 3.1. Establishment of Common PCR Methods

**Figure 1:**
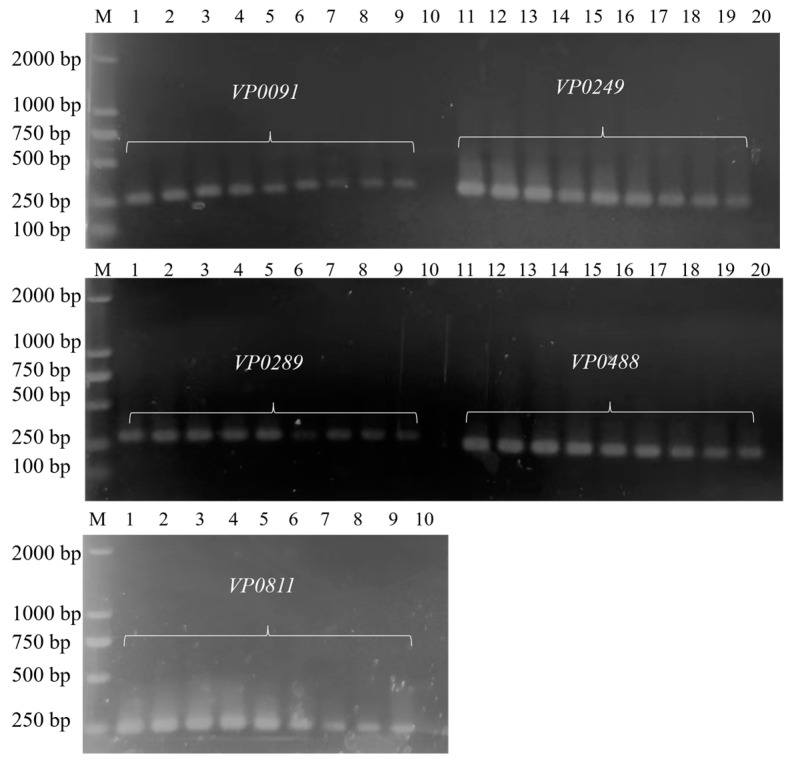
Establishment of a common PCR method and primer screening. M: DL2000 DNA Marker; lanes 1–3: 10-fold diluted *V. parahaemolyticus* DNA template; lanes 4–6: 100-fold diluted *V. parahaemolyticus* DNA template; lanes 7–9: 1000-fold diluted *V. parahaemolyticus* DNA template; lanes 10 and 20: negative control.

As shown in Figure 1, the established ordinary PCR method could successfully amplify five targets without false positives. Among them, the band amplified by the target *VP0811* primers had the largest yield and an obvious gradient. Therefore, the target *VP0811* primer pair was subsequently used for PCR amplification. The amplification product of *VP0811* is 244 bp, and it is calculated that its size exceeds 80 nm. In subsequent experiments, when Eva Green and the amplification product are dropped onto the detection substrate, free Eva Green can be combined with the detection hotspot. Eva Green embedded in dsDNA molecules has difficulty accessing the detection hotspot due to the obstruction of DNA molecules.

### 3.2. Construction of the Detection Substrate

**Figure 2:**
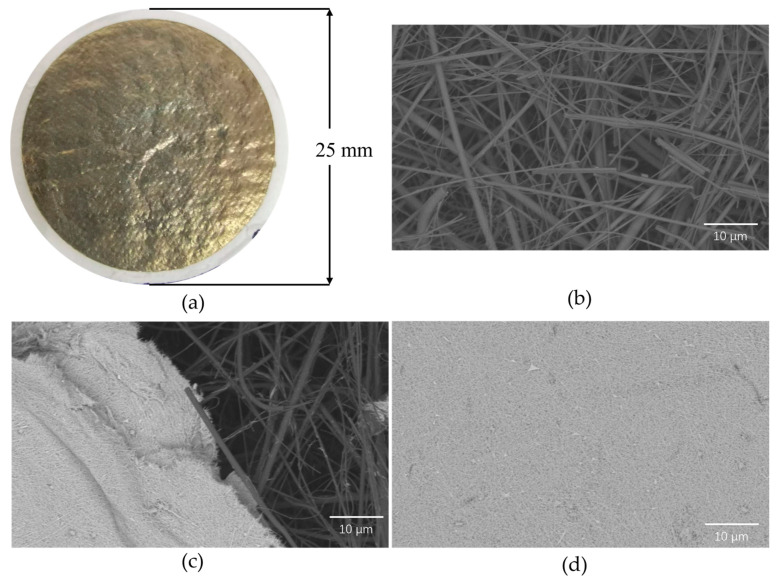
Characterization of the SERS paper-based substrate. (**a**) Image of the paper-based substrate; (**b**) SEM characterization of GFP; (**c**) SEM characterization of AgNWs stacked on GFP; and (**d**) SEM characterization image of AgNWs on the paper-based substrate.

As can be seen in Figure 2a, after vacuum filtration and drying, AgNWs are evenly covered in a thin film on the GFP. The AgNWs are crisscrossed and stacked on each other, which not only form a large number of SERS detection hotspots, but also act as a DNA molecular sieve, allowing the DNA molecules and signaling molecules in the product to be successfully filtered onto the GFP. Figure 2b shows the microscopic morphology of GFP under a scanning electron microscope. The filter paper has larger pores and cannot function as a DNA molecular sieve without being coated with AgNWs. Calculated based on the 244 bp *VP0811* amplified fragment, its size is approximately 85 nm. Considering the nanopores between AgNWs shown in Figure 2c,d, the amplified DNA can be filtered on AgNW substrates, and therefore, it is expected that a low concentration of template will bind less Eva Green, and a high concentration of free signaling molecules will result in relatively high Raman intensity, which is opposite to the number of thermal cycles.

**Figure 3:**
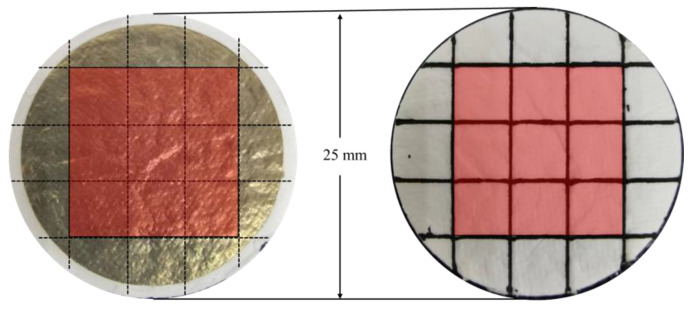
Segmentation of the paper-based substrate.

The paper base needs to be divided. As shown in Figure 3, the glass fiber filter paper with a diameter of 25 mm was divided into 5 mm × 5 mm squares. The part of the circumference that was not completely covered by the AgNWs was removed and the center part of the filter paper with nine squares was kept (marked in red in Figure 3). The filter paper was divided along the dotted lines to obtain nine detection substrates. A total of 10 μL of the PCR amplification product was dropped onto the paper base and dried. There was no obvious change in the base, and the AgNWs did not decompose or detach and could be used for subsequent SERS detection.

### 3.3. Establishment of Detection Methods

**Figure 4:**
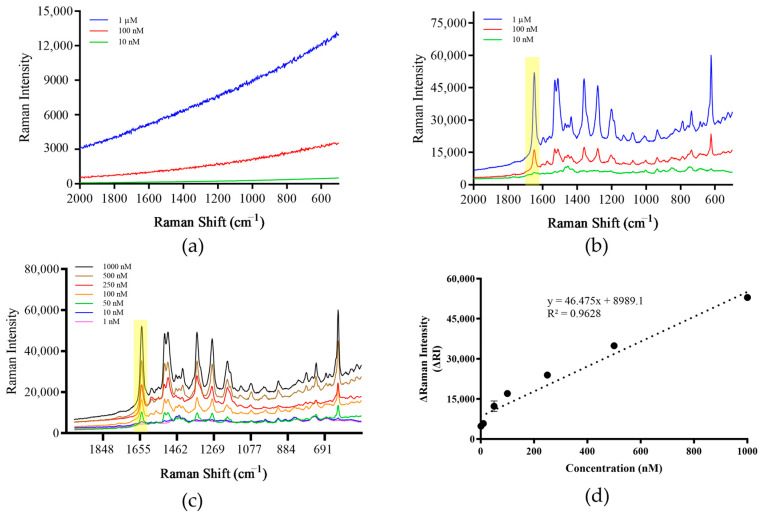
Raman signal detection of Rhod B. (**a**) Direct detection of Rhod B. (**b**) Detection of Rhod B using the paper-based substrate. (**c**) Raman spectrum of gradient concentrations of Rhod B using the paper-based substrate. (**d**) Raman signal standard curve for gradient concentrations of Rhod B. Note: The yellow part of the figure is the most important characteristic peak.

Rhod B is often used as a signaling molecule in SERS. After surface enhancement, it has a characteristic peak at the Raman shift of 1000–1750 cm^−1^. When measured alone, although there was Raman signal, no characteristic peak was produced (Figure 4a). However, when Rhod B was added to the prepared SERS paper-based substrate, the same sample generated characteristic peaks, and as the concentration of Rhod B decreased, the intensity of the characteristic peaks decreased sequentially (Figure 4b). Seven concentration gradients of Rhod B solutions were prepared: 1 μM, 500 nM, 100 nM, 50 nM, 25 nM, 10 nM and 1 nM. After measurement, the Raman signal intensity value of each sample at the Raman shift of 1648 cm^−1^ was taken and a standard curve was drawn. The results show that within the selected concentration range, there is a certain linear relationship between the signal value and sample concentration, and the R^2^ value of the standard curve is 0.963 (Figure 4c,d). Therefore, the prepared paper-based substrate can be used for the detection of Raman signaling molecules.

### 3.4. Screening of Signaling Molecules

**Figure 5:**
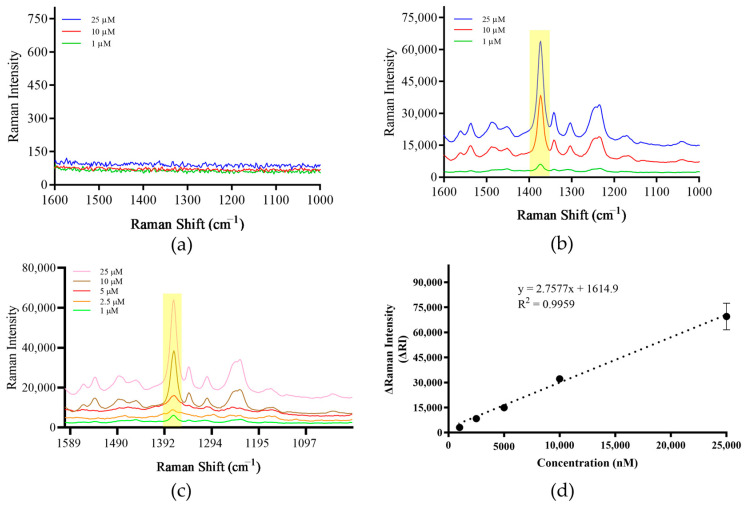
Raman signal detection of Eva Green. (**a**) Direct detection of Eva Green. (**b**) Detection of Eva Green using the paper-based substrate, (**c**) Raman spectrum of gradient concentrations of Eva Green using the paper-based substrate. (**d**) Raman signal standard curve for gradient concentrations of Eva Green. Note: The yellow part of the figure is the most important characteristic peak.

Eva Green is used as a signaling molecule in this study, after surface enhancement, it has a characteristic peak at a Raman shift of 1000–1800 cm^−1^. When measured alone, although there is Raman signal, no characteristic peak is produced (Figure 5a). However, when Eva Green was added to the prepared SERS paper-based substrate, the same sample had characteristic peaks, and as the concentration of Eva Green decreased, the intensity of the characteristic peaks decreased sequentially (Figure 5b). Five concentration gradients of Eva Green solutions were prepared: 25 μM, 10 μM, 5 μM, 2.5 μM, 1 μM. After measurement, the Raman signal intensity value of each sample was taken at the Raman shift of 1373 cm^−1^ and a standard curve was drawn. The results show that within the concentration range, there is a certain linear relationship between signal value and sample concentration, and the R^2^ value of the standard curve is 0.996 (Figure 5c,d). Therefore, the established method can be used for subsequent detection using Eva Green.

**Figure 6:**
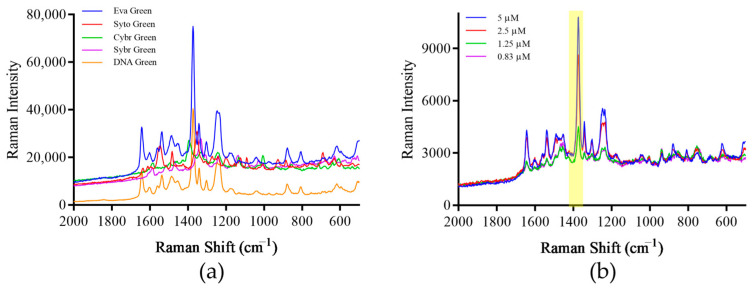
Screening of signaling molecules. (**a**) Screening of signaling molecule types. (**b**) Screening of Eva Green signaling molecule concentrations. Note: The yellow part of the figure is the most important characteristic peak.

In addition to Eva Green, four other DNA dyes were tried, and it was found that five dyes had characteristic peaks at different Raman shifts. Among them, Eva Green had the highest peak at a Raman shift of 1373 cm^−1^, and DNA Green had characteristic peaks at similar positions, while the remaining three DNA dyes had lower peak values at the highest characteristic peaks; so, Eva Green was selected as the optimal signaling molecule for subsequent experiments (Figure 6a). In order to explore the use of Eva Green concentrations, Eva Green was diluted 2-fold, 4-fold, and 6-fold, and mixed with 8 μL of PCR product of the same concentration to detect the Raman signal, so that the final concentration was 5 μM, 2.5 μM, 1.25 μM, and 0.83 μM. The results showed that after mixing with the PCR product, the signal intensity reported by Eva Green was significantly lower than that of the original solution, indicating that the combination of the PCR product with Eva Green resulted in its consumption, and the content of free signaling molecules in the liquid decreased, resulting in weakened Raman signals (Figure 6b). For the same amount of PCR product, when the concentration of Eva Green was reduced, the intensity of the Raman signal also gradually decreased. This phenomenon was detrimental to the improvement of the detection efficiency of the method. Therefore, the final concentration of Eva Green was determined to be 5 μM.

### 3.5. PCR Amplification Time Optimization Results

**Figure 7:**
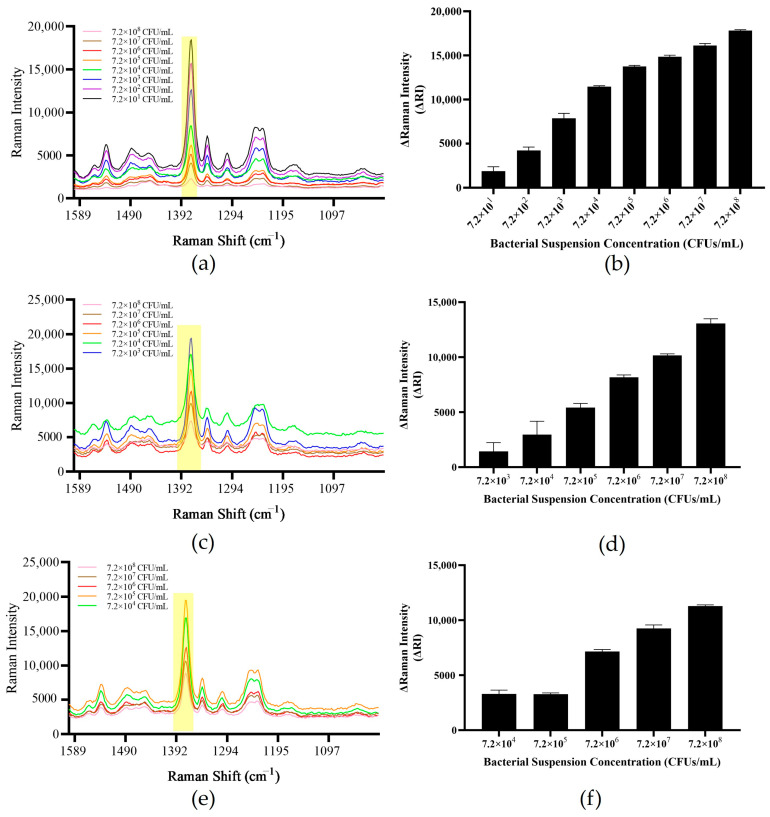
PCR amplification time optimization. (**a**,**b**) Method detection limit at 30 PCR cycles. (**c**,**d**) Method detection limit at 20 PCR cycles. (**e**,**f**) Method detection limit at 10 PCR cycles. Note: The yellow part of the figure is the most important characteristic peak.

The PCR amplification time determines the detection time of the entire method. When the amplification cycle number is 10 cycles, the PCR amplification time is about 10 min, and the whole method detection time is 15 min. As the number of PCR cycles increased from 10 to 30, the detection time also increased from 15 min to 35 min. After 10 cycles of amplification, it was observed that the method could only detect templates with a concentration range of 7.2 × 10^4^–7.2 × 10^8^ CFUs/mL, and when the template concentration was 7.2 × 10^4^ CFUs/mL and 7.2 × 10^5^ CFUs/mL, the ΔRI values of the two systems were similar; so, when the number of amplification cycles is 10 cycles, the detection capability of the method is limited. When the cycle number was increased to 20 cycles, the detection capability of the method was improved, and the target bacteria could be detected at a concentration of 7.2 × 10^3^ CFUs/mL. However, the detection limit had not yet reached a satisfactory level for on-site detection. Therefore, we continued to increase the number of amplification cycles to 30 cycles. At this time, 7.2 × 10^1^ CFUs/mL of bacterial suspension could be detected by the system. This sensitivity meets the needs of on-site detection. Continuing to increase the number of amplification cycles will increase the detection time, and so the number of PCR amplification cycles of the final method was 30 cycles and the overall detection time could be controlled within 40 min (Figure 7).

**Figure 8:**
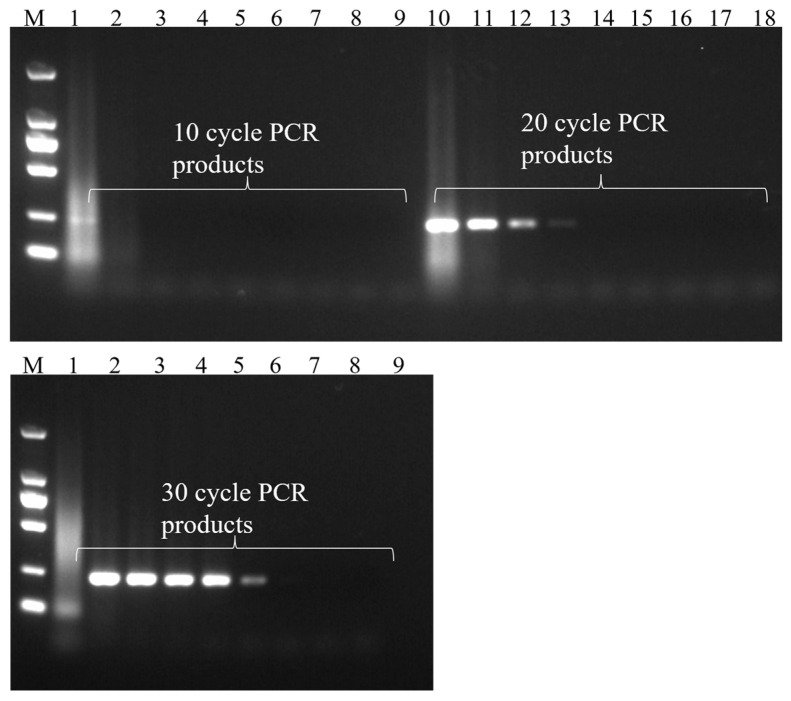
Gel electrophoresis images of PCR products at different cycle numbers. DL2000 DNA Marker; lanes 1–9 and 10–18: gradient dilutions of the *V. parahaemolyticus* DNA template.

Compared with the method established in this study, gel electrophoresis analysis was performed on PCR products at the same cycle number (Figure 8). When the number of PCR cycles was 10 cycles, only the PCR products of the templates with two concentrations of 7.2 × 10^7^ CFUs/mL and 7.2 × 10^8^ CFUs/mL had fuzzy bands in the agarose gel. When the number of PCR cycles was 20 cycles, the PCR products of four templates with concentration of 7.2 × 10^5^–7.2 × 10^8^ CFUs/mL produced clear bands in the agarose gel. However, at this time, the gel electrophoresis method could only detect 7.2 × 10^5^ CFUs/mL of the bacterial suspension template, which was two orders of magnitude lower than the sensitivity of 7.2 × 10^3^ CFUs/mL of the method established in this study. When the number of PCR cycles was 30 cycles, the PCR products of six templates with concentration of 7.2 × 10^3^–7.2 × 10^8^ CFUs/mL produced clear bands in the agarose gel, but at this time, the detection limit of the gel electrophoresis method was only 7.2 × 10^5^ CFUs/mL, which was two orders of magnitude lower than the sensitivity of 7.2 × 10^1^ CFUs/mL of the method established in this study. The PCR-SERS method established in this study eliminates the time-consuming and laborious electrophoresis operation and improves the detection sensitivity by two orders of magnitude.

### 3.6. Method Sensitivity Assessment

**Figure 9:**
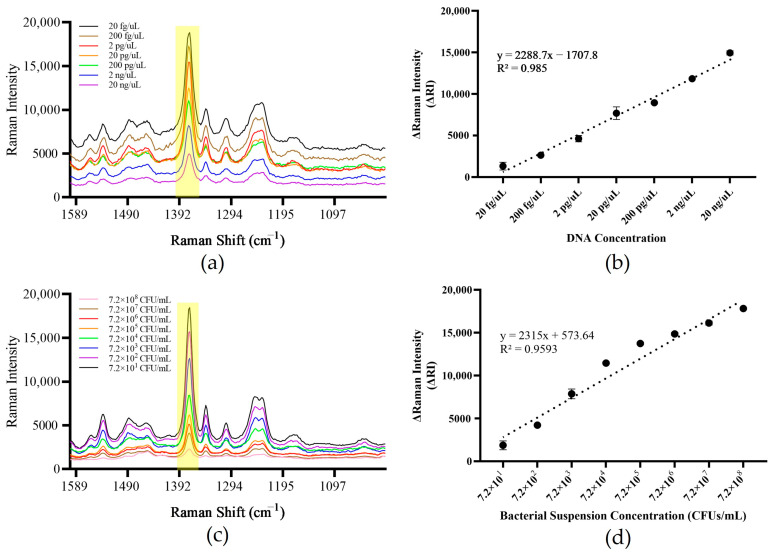
Method sensitivity evaluation. (**a**,**b**) DNA sensitivity and standard curve of the established method. (**c**,**d**) Bacterial suspension sensitivity and standard curve of the established method. Note: The yellow part of the figure is the most important characteristic peak.

For the DNA template, when the template concentration was 20 fg/μL–20 ng/μL, the system generated positive signals. When the template concentration was lower than 20 fg/μL and reached 2 fg/μL, the system ΔRI was less than zero and reported as undetected. The standard curve was drawn with the ΔRI value as the ordinate and the template concentration as the abscissa, showing a good linear relationship, with an R^2^ value of 0.985 (Figure 9a,b). For the bacterial suspension template, when the template concentration was 7.2 × 10^1^–7.2 × 10^8^ CFUs/mL, the system generated positive signals. When the template concentration was lower than 7.2 × 10^1^ CFUs/mL and reached 7.2 CFUs/mL, the system ΔRI value was less than zero and reported as undetected. The standard curve was drawn with the ΔRI value as the ordinate and the template concentration as the abscissa, showing a good linear relationship, with an R^2^ value of 0.959 (Figure 9c,d). The above results show that the method has a lower detection limit, which can reach 20 fg/μL for the DNA template and 7.2 × 10^1^ CFUs/mL for the bacterial suspension template.

### 3.7. Method Specificity Assessment

**Figure 10:**
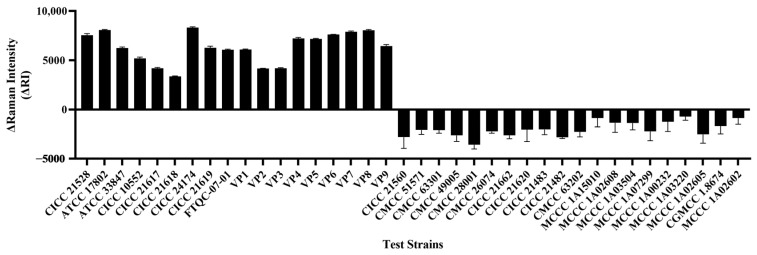
Specificity evaluation results.

The specificity of the method was evaluated using 38 strains (Table 2). Among them, 18 were *V. parahaemolyticus* strains, all of which produced positive signals in the system, and their ΔRI values were all greater than zero. For 20 negative strains, their ΔRI values were all less than zero, all of which was reported as undetected. Therefore, the method has good specificity and can only detect *V. parahaemolyticus*, but cannot detect common foodborne pathogenic bacteria and non-*V. parahaemolyticus* strains (Figure 10).

### 3.8. Detection of Artificially Contaminated Samples

**Figure 11:**
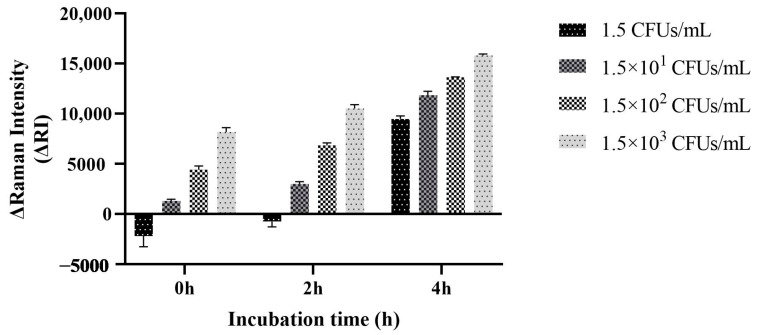
Test results for artificially contaminated samples.

For artificially contaminated samples under uncultured conditions, the ΔRI value of the sample contaminated with 1.5 CFUs/mL *V. parahaemolyticus* was less than zero and reported as undetected, while samples with concentrations of 1.5 × 10^1^–1.5 × 10^3^ CFUs/mL generated positive signals, and the ΔRI values of the system between templates with different gradient concentrations increased sequentially. After 2 h of enrichment culture, the ΔRI value of the sample contaminated with 1.5 CFUs/mL *V. parahaemolyticus* was less than zero and was still determined to be undetected. The ΔRI value of the sample with concentrations of 1.5 × 10^1^–1.5 × 10^3^ CFUs/mL increased. When the enrichment culture time was 4 h, the ΔRI values corresponding to each concentration of the template increased, and the ΔRI value of the sample contaminated with 1.5 CFUs/mL *V. parahaemolyticus* was close to 10,000, which was judged to be a strong positive signal. Therefore, when the enrichment culture time reached 4 h, the target strain with an initial contamination amount of 1.5 CFUs/mL could be detected (Figure 11). Therefore, in the subsequent application of the method, it is recommended to enrich and culture the samples to be tested to increase the detection rate, and the enrichment culture time is recommended to be 4 h.

### 3.9. Determination of the Actual Sample Recovery Rate

Four commercially available actual samples inoculated with the target strain can all produce positive signals, with recovery rates between 114.03% and 195.10%, and RSD values between 1.05 and 4.49%, which are within the acceptable range (Table 3). Although the method can complete the qualitative detection and relative quantitative detection of samples, when applied to the detection of actual samples, the evaluation results will be higher to a certain extent, mainly due to the amplification of dual signals, subtle PCR amplification errors, or pull-down. The detection fluctuations of the Mann signal will cause large errors in the detection results, which is also a shortcoming of this method. However, compared with ordinary PCR, the established method can not only be qualitative but also provide relative quantitative capabilities. Therefore, follow-up research on the stability of the method can be tried and improved.

## 4. Discussion

The SERS technology currently used in microbial detection is generally divided into two types, label-free SERS detection and labeled SERS detection, namely, direct detection and indirect detection. In direct detection, the nano-substrate and microorganisms adsorb each other through electrostatic attraction, and the SERS spectrum of the target molecule can be directly obtained. Raman tags for indirect detection consist of nano-substrates and Raman signaling molecules, through aptamers [19], antibodies [20], phages [21], etc. A highly specific capture element is used to capture the target bacteria and connect it to a Raman tag to indirectly quantify the microbial concentration using the characteristic peaks of the Raman signaling molecules [22]. The application of surface-enhanced Raman spectroscopy (SERS) in food quality and safety analysis is growing rapidly, especially throughout the food chain, including from farm to fork, food processing, and the detection of genetically modified and novel foods. SERS technology is considered a tool with high potential in the field of food testing due to its high sensitivity, fast response, non-destructiveness, and simple sample preparation [23]. For example, Jin et al. [24] proposed a method to simultaneously detect low concentrations of potassium sorbate and lead in matsutake based on SERS and fluorescence spectroscopy, which can accurately predict the residual amounts of preservatives and heavy metals in matsutake, providing a reference for other food safety testing. Zhang et al. [25] found that the combination of SERS and machine learning algorithms can effectively and quickly detect the content of Auramine O in various traditional Chinese medicines.

In most cases, a single plasmonic nanoparticle cannot provide a sufficient enhancement effect for practical applications of SERS. When two nanoparticles are close enough, a considerable SERS enhancement effect can be produced in the gap, which exhibits extremely high electric fields. Spatially localized volumes that enhance and produce strong SERS signals are called hot spots [26]. For example, Tang et al. [27] used a silver nanoparticle composite polyacrylonitrile (Ag-NPs@PAN) to construct a three-dimensional SERS substrate with higher sensitivity and greater “hotspot” density to capture and amplify the SERS signal of carbendazim, achieving rapid and accurate quantitative detection of carbendazim. Hot spots usually exist in the gaps between nanoparticle aggregates. This study promotes the formation of random, dense, and uniform detection hot spots by excessively attaching silver nanowires to the glass-fiber filter paper, thus maximizing the elimination of result errors caused by uneven detection substrates.

Secondly, since the SERS mechanism requires molecules to stay close to the metal surface to generate a strong SERS signal, the diffusion of molecules on the surface and in the solution may cause the SERS signal to change over time, especially when the target molecule has a weak affinity for the surface or the concentration is ultra-low, and the signal instability is more obvious [28]. When the molecule moves out of the hotspot, the Raman signal of the molecule cannot be effectively enhanced, and the detected signal is mainly contributed by the substrate, solvent, impurities or free molecules, or fluorescence, becoming noise in the SERS spectrum [29]. In this study, the preparation of the detection substrate and the detection of the sample required two drying and fixation processes, which effectively avoided signal fluctuations caused by the floating state of the signaling molecules.

Finally, through primer design, the PCR amplification product has a suitable size and cannot pass through the AgNW molecular sieve and is intercepted on the paper base. Due to the spatial effect of DNA molecules, the Eva Green embedded in it is isolated from the detection hotspot on the substrate, and so the Raman signal is very sensitive to the content of DNA molecules in the amplification product. In this case, the combination of PCR and SERS produced a satisfactory and stable effect, and the detection results of pure bacterial culture and template DNA showed that there was a good linear relationship between the Raman signal and the template concentrations. Compared with the conventional PCR method, this method does not need to detect the amplification results by gel electrophoresis, and so the detection time is shortened. Additionally, SERS can improve the reaction sensitivity up to 7.2 × 10^1^ CFUs/mL. Moreover, the method provides a relative quantitative ability that cannot be achieved by conventional PCR. In addition, this detection method provided a reference for the construction of a DNA detection platform based on SERS. We also compared the detection limit of the constructed method with other rapid detection methods for *V. parahaemolyticus* (Table 4). In newly published research, two biosensor-based detection methods have high detection limits below 10 CFUs/mL. At the same time, the combination of multifunctional composite magnetic materials and magnetophoretic chromatography technology for SERS detection also shows a higher detection limit. The paper-based sensor method based on FRET and the new strategy based on lanthanide metal–organic frameworks and aptamers show detection limits similar to that in this study, and the detection limit of the ordinary PCR method combined with PMA is much lower than that of this study. Therefore, the detection limit of the method constructed in this study is within the acceptable range.

As with any analytical method, PCR-SERS established in this study has some drawbacks. First of all, the target used in this study is a newly screened target. Whether it is related to the pathogenicity of the strain requires in-depth study. Virulence genes commonly used for the detection of *V. parahaemolyticus*, such as *tdh* and *trh*, have been detected in environmental isolates. The rate is low. For the time being, this study cannot distinguish the two types of strains based on a single gene. The difficulty of this study is the preparation of the detection substrate and the establishment of the detection method. Therefore, on the basis of this study, virulence genes and specific targets can be located using a dual PCR system to distinguish environmental strains from virulence strains. Secondly, the popularity of Raman spectrometers is not high. They are often found in some testing institutions or universities in developed areas, and do not have testing conditions in underdeveloped areas or some universities with backward resources. At this point, the popularity rate of Raman spectrometers is not as good as that of ordinary PCR machines. Then, the cost of Raman spectrometer is relatively high, and the operator requires high professionalism. Finally, one of the major obstacles that DNA sensors based on SERS need to overcome is the lack of a certain repeatability of their detection signals. Due to the particularity of the Raman signal, the non-uniform size and shape of the nanoparticles will cause the Raman signal to shift up or down. Small changes in the sample shape will also cause changes in the Raman signal. Therefore, this effect greatly hinders the promotion and application of Raman spectroscopy in detection work that requires precision [37].

So far, there are no reports on the clinical application of SERS-DNA sensors. The fundamental reason is that the SERS-DNA sensor has defects in stability and sensitivity in most cases [38]. In this study, the combination of PCR and SERS methods was applied to foodborne pathogenic bacteria to prove its potential in detecting trace amounts of DNA in food samples. In order to promote the application of SERS-DNA sensors and stimulate the development of more SERS-DNA sensors, it is necessary to develop new, highly active, stable, and highly reproducible signaling molecules or substrates that can be used for SERS measurements. In order to improve the sensitivity and reproducibility of SERS detection methods, in the past nearly 50 years, a large amount of research has been devoted to the preparation of nanomaterials with high SERS activity. In addition to Ag materials, AuNPs are suitable for excitation beyond 600 nm, and due to their excellent biocompatibility, they are often used in intracellular or in vivo research [39]. Qiu et al. [40] made flexible materials by assembling β-cyclodextrin-modified gold nanoparticles on a polytetrafluoroethylene film coated with a perfluorinated liquid. The SERS substrate enables the in situ detection and identification of various PAH residues on fruits and vegetables. In addition, more and more reports indicate the use of multi-component nanoparticles (including core-shell nanoparticles and alloy nanoparticles) as SERS substrates to achieve optimized performance; especially for core-shell nanostructures, the existence of extremely small gaps will greatly enhance the Raman signals of molecules in the gaps and adsorbed on the shell [41]. Although the relevant enhancement mechanisms have yet to be explored, core-shell nanostructures have been shown to emit signals even at the single-particle level, and compared with nanoparticle aggregates, core-shell nanostructures have very uniform enhancement effects without affecting sensitivity [42]. At the same time, in order to give full play to the high detection sensitivity of SERS and maintain the reliability of measurement, correct measurement conditions are crucial. When selecting the excitation wavelength for SERS measurement, the aggregation state of nanomaterials needs to be considered. For individual nanoparticles, the UV absorption peak position should be referenced, while longer wavelengths should be used for nanoparticles in an aggregated state or assembled on a solid substrate [43]. Therefore, in addition to focusing on the development of emerging detection strategies, researchers should also focus on the discovery of new materials to provide more basic data for the establishment of relevant methods. Although SERS technology has not yet been used for actual detection, with the continuous optimization of SERS substrates, the emergence of numerous portable and handheld Raman spectrometers, and the advancement of coupling with other technologies, SERS is expected to become a reliable and routine technology for rapid detection.

## 5. Conclusions

In summary, in this study, a combination of GFP and AgNWs was used to construct a SERS paper-based substrate, and the DNA chimeric dye Eva Green was used as a signaling molecule to determine the initial template concentration by measuring the signal intensity at a special Raman shift. The detection time of this method can be controlled within 40 min, including 30 min of PCR amplification and a 10 min SERS measurement time. The established method has high sensitivity and can accurately detect a DNA template of 20 fg/μL–20 ng/μL and bacterial suspension template of 7.2 × 10^1^ CFUs/mL–7.2 × 10^8^ CFUs/mL, which is two orders of magnitude higher than conventional gel electrophoresis. The method is applied to the detection of artificially contaminated samples, and the target bacteria with an initial inoculation amount of 1.5 CFUs/mL can be detected at a bacterial enrichment time of 4 h, providing a powerful means for the surveillance of related foodborne diseases.

## Figures and Tables

**Table 1:** Primer information.

Gene	Sequences (5′-3′)	Product Length (bp)
*VP0091*	F: TCAGGCGATGATTGTGCTGT	270
	R: GCGAAATTCATTACCCCCGC	
*VP0249*	F: GGCCGATTTTCATCGTCAGC	258
	R: GATTCATGGTATCCGCCCGT	
*VP0289*	F: GACTGGGTGATCGGCTTTGA	289
	R: CGAGGTAAGCCGCAATTTGG	
*VP0488*	F: GTGAACTGGCAACAAGGCAG	252
	R: AGTGCGAGTTTCCAAGTCCC	
*VP0811*	F: AACAAGCCGAGTTCTAGCCC	244
	R: GTCTCGCCTTCAGAGCCAAT	

**Table 2:** Specific evaluated strains and results.

Strain Category	Strain No.	Detection Result
*Vibrio parahaemolyticus*	CICC 21528	+
*Vibrio parahaemolyticus*	ATCC 17802	+
*Vibrio parahaemolyticus*	ATCC 33847	+
*Vibrio parahaemolyticus*	CICC 10552	+
*Vibrio parahaemolyticus*	CICC 21617	+
*Vibrio parahaemolyticus*	CICC 21618	+
*Vibrio parahaemolyticus*	CICC 24174	+
*Vibrio parahaemolyticus*	CICC 21619	+
*Vibrio parahaemolyticus*	FTQC-07-01	+
*Vibrio parahaemolyticus*	VP1-9	+
*Enterobacter sakazakii*	CICC 21560	-
*Shigella flexneri*	CMCC 51571	-
*Bacillus cereus*	CMCC 63301	-
*Proteus mirabilis*	CMCC 49005	-
*Micrococcus luteus*	CMCC 28001	-
*Staphylococcus aureus*	CMCC 26074	-
*Listeria monocytogenes*	CICC 21662	-
*Pseudomonas fluorescens*	CICC 21620	-
*Salmonella Typhimurium*	CICC 21483	-
*Salmonella enteritidis*	CICC 21482	-
*Bacillus pumilus*	CMCC 63202	-
*Vibrio jasicida*	MCCC 1A15010	-
*Vibrio cholerae*	MCCC 1A02608	-
*Vibrio diabolicus -*	MCCC 1A03504	-
*Vibrio anguillarum -*	MCCC 1A07299	-
*Vibrio harveyi*	MCCC 1A00232	-
*Vibrio alginolyticus -*	MCCC 1A03220	-
*Vibrio campbellii*	MCCC 1A02605	-
*Vibrio vulnificus -*	CGMCC 1.8674	-
*Vibrio mimicus*	MCCC 1A02602	-

**Table 3:** Actual sample recovery rate measurement results.

Samples	Added (CFUs/mL)	Recovered (CFUs/mL)	Recovery %	RSD %
oysters	5000	5701.416	114.03	4.49
clams	5000	5628.17	112.56	2.75
cod	5000	6771.934	135.48	2.90
river shrimp	5000	9755.377	195.10	1.05

**Table 4:** Comparison of different detection methods for *Vibrio parahaemolyticus*.

Detection Method	Detection Limit (CFUs/mL)	Ref.
FRET-based paper sensor with a smartphone	67	[30]
Biosensing strategy mediated by DNAzyme–ferrocene-triggered click chemistry	6	[31]
PMA-mPCR	1.05 × 10^3^	[32]
Phagomagnetic separation–ATP bioluminescence (PhMS-BL)	78	[33]
Dual-mode colorimetric-electrochemical biosensor	5	[34]
Visual analysis strategy based on lanthanide metal–organic frameworks and a FAM-aptamer	100	[35]
Colorimetric and SERS dual-mode detection using composite magnetic materials combined with magnetophoretic chromatography technology	7	[36]
PCR-SERS	72	This work

## Data Availability

The original contributions presented in the study are included in the article, further inquiries can be directed to the corresponding authors.
